# Metformin, Oxidative Stress, and Infertility: A Way Forward

**DOI:** 10.3389/fphys.2018.01722

**Published:** 2018-11-29

**Authors:** Rehana Rehman, Syed Hani Abidi, Faiza Alam

**Affiliations:** ^1^Department of Biological and Biomedical Sciences, Aga Khan University, Karachi, Pakistan; ^2^Department of Physiology, University of Karachi, Karachi, Pakistan

**Keywords:** antioxidants, female infertility, oxidative stress, SIRT1, metformin

## Introduction

Infertility is a significant public health problem and its diagnosis and treatment are stressful, invasive and costly. Impaired fecundity is a growing health concern worldwide. Fertility predominantly depends on the maintenance of the quantity and quality of the ovarian reserve, which is determined by the extrinsic and intrinsic factors. The reserve declines with consumption of the follicle as age increases and by the imbalance in the redox activity.

Cellular redox activity is a normal mechanism of the male and female reproductive system but its inequilibrium affects the fertility by hindering attainment and maintenance of oocyte developmental potential during *in vivo* processes including ovarian aging. Oxidative stress (OS) has been associated with decreased female fecundity in animal and *in vitro* models, but no studies to date have directly assessed the relationship in women. Exposures associated with OS and pregnancy outcomes have so far been studied in relation to Poly Cystic Ovarian Syndrome (PCOS) only. Sirtuin (silent information regulator, SIRT), family of NAD dependent enzymes is evolving as main regulator of OS. They are known to repair cells damaged due to OS by stimulating the expression of antioxidants and thus preventing dysfunction of the ovarian cells. SIRT1 signaling initiates a positive ovarian cells response by catalyzing an enzymatic reaction between nicotinamide and the acetyl group of the substrate, transferring it to cleave NAD, generating a unique metabolite, O-acetyl-ADP ribose (Pillarisetti, 2008). Alterations in cellular NAD levels, or the NAD-NADH ratio, are the primary mechanisms controlling SIRT1 expression and activity(Guarente and Picard, 2005).

As far as the impact of Os on oocyte is concerned; under influence of various external and internal factors, when the oocytes start aging and generating advanced glycation end products (AGEs) in the ovarian microenvironment, there is a trigger for the generation of intracellular reactive oxygen species ROS by down regulation of NAD(P)H oxidase, mitogen-activated protein kinases (MAPKs), and the transcription factor, nuclear factor kappa B (NF-.B) (Lander et al., 1997; Xu and Kyriakis, 2003) leading to proinflammatory milieu and an increase in OS (Mohamed et al., 1999; Schmidt et al., 2001). Lipid peroxidation during OS is capable of commencing cyclic ROS dissemination via direct damage to mtDNA (mitochondrial DNA) and proteins. With increase concentrations of ROS within the oocytes, mitochondria lose their potential to culminate the state of auto-oxidation by decreased production of ATP from the electron transport chain (ETC), thus aggravating damage to mitochondrial functional components.

Mitochondria being a key player in calcium regulation in the oocytes for generation of the main bulk of ATP for the processes like apoptosis and completion of meiosis including spindle assembly, under the influence of ROS within the ovaries is considered as the main cause for chromosomal segregation disorders, maturation, and fertilization failures, or oocyte/embryo fragmentation. Presence of systemic and follicular oxidative stress markers in patients of endometriosis have also been identified (Takeuchi et al., 2005). Maintenance of the oxidative environment within the oocytes is an essential process, which needs to be guarded and carried out by the regulators.

Significant inhibition of SIRT1 mRNA has also been observed with increase in ROS, compromising the nuclear maturation and the mitotic spindles of the maturing oocytes.

An independent study conducted in Pakistan suggests that functions of SIRT1 are affected by various post-transcriptional activities; increased by phosphorylation, while glycosylation decreased it (Hanover et al., 2010). It utilizes unique pathways; its glycosylation occurs through the hexoseamine-signaling pathway (Hanover et al., 2010), furthermore, SIRT1-dependent gluconeogenesis is also intermediated by changes in the levels of NAD and pyruvate instead of employing gluconeogenic regulatory hormones (Rodgers et al., 2005). The crosstalk between various post-translational modifications regulates the proteins functionally that control the transcriptional activity of various genes(Hoessli et al., 2013). Addition of O-GlcNAc during the process of aging and development of diabetes weakens the activity of SIRT.

**Figure 1:**
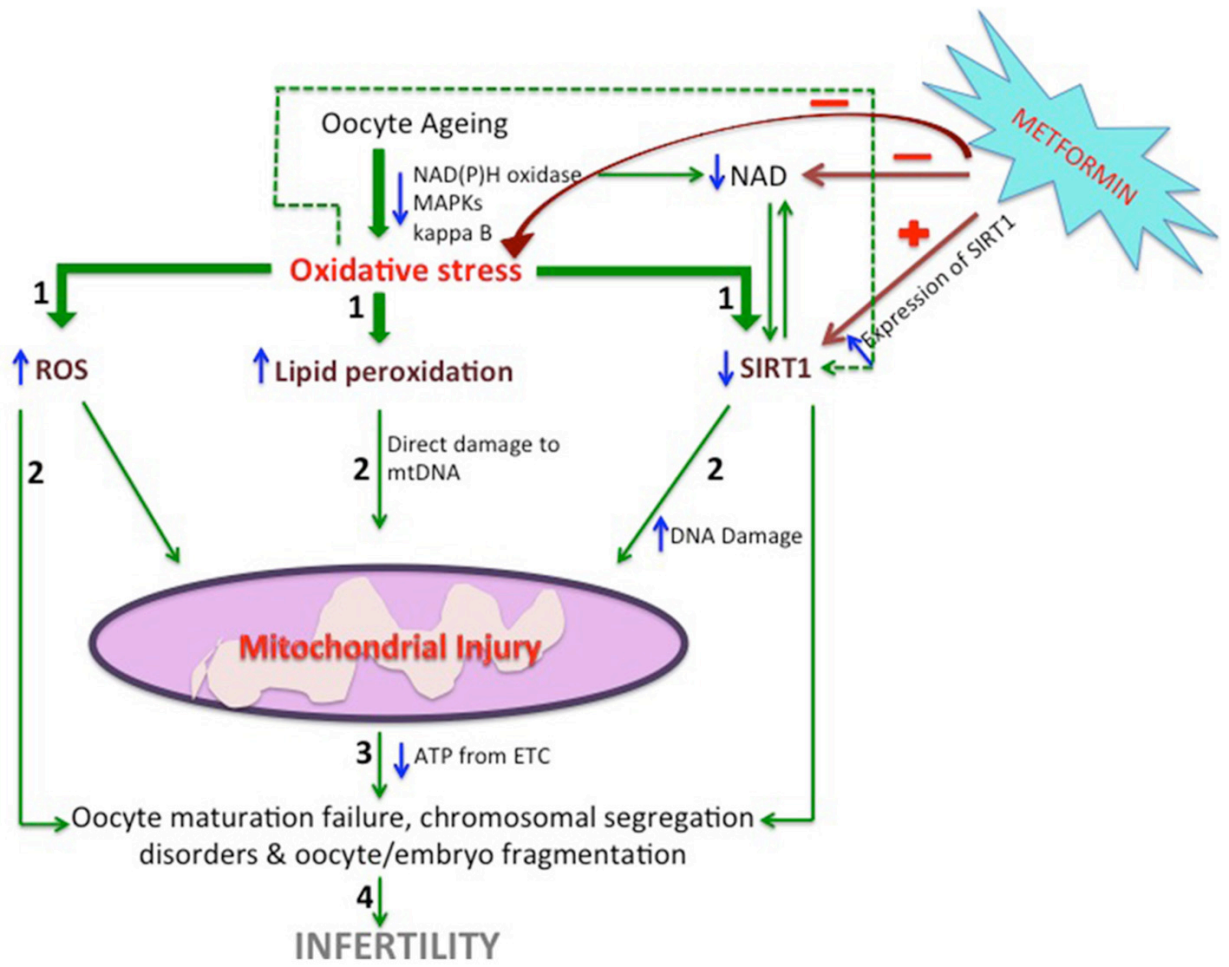
A hypothetical view of the possible mechanisms by which Metformin helps in maintaining the microenvironment of the granulosa cells. With advancing age of the oocytes, development of oxidative stress takes place which leads to imbalance between the oxidants and the antioxidants thus elevating reactive oxygen species, lipid peroxidation and simultaneously decreasing the expression of SIRT1 via decreased NAD/NADPH ratio (STEP1). These changes lead to mitochondrial dysfunction by direct injury to the mitochondrial DNA (STEP2) depleting ATP synthesis by the electron transport chain (STEP3). Oocyte maturation failure, chromosomal segregation disorders & oocyte/embryo fragmentation occurs (STEP4) as a result causing infertility. Metformin moderates the expression of SIRT1 directly and by regulating the NAD/NADPH ratio which also in turn increases the concentration of SIRT1. It is also believed to buffer the ROS by increasing the concentration of Glutathione. NAD(P)H, Nicotinamide adenine dinucleotide phosphate; MAPKs, mitogen-activated protein kinase; ROS, reactive oxygen species; mtDNA, mitochondrial DNA; SIRT, Sirtuin; ATP, Adenosine-5-triphosphate.

Metformin; (generic name; Dimethylbiguanide), widely used hypoglycemic drug utilizing its capability of inhibiting hepatic gluconeogenesis. It is recently known to inhibit cellular respiration carried out by the mitochondria in the hepatic cells, however its effect is dose-dependent and less potent on the intact cells. Among the cell signaling pathways capable of mitochondrial regulation, metformin contribute to increase in the glutathione content, thus providing aid in encountering the ROS (Figure 1). Metformin induces increase in NAD/NADH ratio by inducing increased expression of NAMPT mRNA and protein expression, resulting in enhanced SIRT1 expression and activity in a dose-dependent fashion (Caton et al., 2010).

Metformin has been a choice in treating PCOS as a single as well as in adjuvant therapy. Despite being successfully administered postulating its classical effect on insulin sensitivity on the cell surface yet its exact action is ill-defined. It induces rise in NAD/NADH ratio by triggering increased expression of NAMPT mRNA, resulting in enhanced SIRT1 expression and activity in a dose-dependent fashion. However pathway proposed in another recent study is it's action on SIRT1 expression by increasing a rate-limiting cytokine in NAD biosynthesis, visfatin(Reverchon et al., 2013).

A previous meta-analysis published on 2006 could not verify the role of Metformin in gonadotropin ovulation induction (Costello et al., 2006). However some studies have validated the benefits of Metformin treatment in PCOS patients by reducing risk of miscarriage and of implantation failure in IVF cycles (Palomba et al., 2013). This suggests that Metformin offer much more than what is known. During the periods of stress, metformin regulates the cellular metabolic machinery and ensure cell survival by regulating the co-ordination among several different age related transcription factor pathways previously mentioned.

Insights into the role of Metformin in decreasing OS in the gynecologic environment might be helpful in understanding the potential of Metformin as an inexpensive and non-invasive therapy for increasing fertility and decreasing the cost of treatment especially in couples with options like *in vitro* fertilization (IVF) & intracytoplasmic sperm injection (ICSI) that are expensive and still have a limited success rate of 25–30%. Studies do support conservation of fertility treatments that instigate a SIRT1 mediated decrease in ROS due to aging and OS.

On a larger scale, studies do support conservation of fertility treatments that instigate SIRT1 mediated decrease in ROS under the circumstances of aging and OS. However, no human study refers to the role of MetF during OS in presence of SIRT1 genetic polymorphism of infertile females. Further comprehensive studies are needed to verify this function of MetF in infertility. This pioneering study, therefore, aims to identify the role of MetF on oxidative stress with existing SIRT1 Polymorphism in infertile cases where SIRT1 polymorphism is associated with aging and OS.

## Hypothesis

We hypothesized that Metformin buffers the increased OS in presence of SIRT1 polymorphism by regulating the NAD biosynthesis and increasing the expression of SIRT1, thus improving the reproductive microenvironment in infertile females. Oxidative environment of the granulosa cells in presence of SIRT1 polymorphism might be improved by the use of Metformin.

## Recommendations

Genetic studies along with cell lines studies are stipulated in order to identify the authentic role of Metformin in decreasing OS in the gynecologic microenvironment. This might surface an inexpensive and non-invasive therapy for increasing fertility and decreasing the cost of treatment especially in couple that are left-with no options other than *in vitro* fertilization (IVF) & intracytoplasmic sperm injection and still have a negative outcome. Metformin could be incorporated as a part of clinical trials in polymorphic infertile patients, if proven could lead to successful outcomes by cost-effective treatments.

## Author contributions

FA and RR conceived and designed the experiments. Manuscript writing was done by RR, SA, and FA.

### Conflict of interest statement

The authors declare that the research was conducted in the absence of any commercial or financial relationships that could be construed as a potential conflict of interest.
